# *Cryptococcus gattii*: An Emerging Cause of Fungal Disease in North America

**DOI:** 10.1155/2009/840452

**Published:** 2009-05-25

**Authors:** Ashwin Dixit, Scott F. Carroll, Salman T. Qureshi

**Affiliations:** ^1^Centre for the Study of Host Resistance, McGill University, Montreal, QC, Canada H3G 1A4; ^2^Department of Microbiology and Immunology, McGill University, Montreal, QC, Canada H3A 2B4; ^3^Department of Human Genetics, McGill University, Montreal, QC, Canada H3A 1B1; ^4^Department of Medicine, McGill University, Montreal, QC, Canada H3G 1A4

## Abstract

During the latter half of the twentieth century, fungal pathogens such as *Cryptococcus neoformans* were increasingly recognized as a significant threat to the health of immune compromised populations throughout the world. Until recently, the closely related species *C. gattii* was considered to be a low-level endemic pathogen that was confined to tropical regions such as Australia. Since 1999, *C. gattii* has emerged in the Pacific Northwest region of North America and has been responsible for a large disease epidemic among generally healthy individuals. The changing epidemiology of *C. gattii* infection is likely to be a consequence of alterations in fungal ecology and biology and illustrates its potential to cause serious human disease. This review summarizes selected biological and clinical aspects of *C. gattii* that are particularly relevant to the recent North American outbreak and compares these to the Australian and South American experience.

## 1. Introduction

Although less than 500 of the estimated 1.5 million species of fungi pose a threat to humans and animals [1, 2], the prevalence of fungal infections has risen over the last century due to a progressive increase in the number of debilitated individuals. Impaired immunity against fungi may result from one or more factors including malignancy, advanced or severe comorbid disease, or the use of cytotoxic drugs and broad-spectrum antibiotics. Species of *Candida* remain the most common cause of invasive yeast infection; however, opportunistic filamentous fungi such as *Aspergillus* spp, *Fusarium* spp, *Scedosporium* spp, *Penicillium* spp, and the zygomycota are becoming more prevalent in oncology and transplant centers [3, 4]. Human disease resulting from environmental exposure to the basidiomycetous yeast *Cryptococcus neoformans* increased significantly since the onset of the HIV epidemic and continues to be common among individuals that do not have access to effective antiretroviral therapy [5, 6]. *C. gattii*, a closely related species that was traditionally associated with tropical and subtropical climates, is now gaining prominence as a cause of human and veterinary disease in North America. While most clinical cases have occurred among generally healthy individuals that reside in the Pacific Northwest, a few tourists or visitors to the region have also been affected. The purpose of this review is to summarize important biological and clinical characteristics of *C. gattii* that are relevant to the understanding of human disease caused by this emerging fungal pathogen.

## 2. Identification

*C. neoformans* was first isolated in 1894 from fermented peach juice by the Italian Francesco Sanfelice [7]. Since that time, this organism has been recovered from numerous locations throughout the world where its main ecological niche is soil, particularly in association with pigeon excreta [8–10]. Cryptococci grow as unicellular, encapsulated cells in the asexual state or as basidiomycetous filaments in the sexual state [1, 11]. Infection due to this opportunistic fungus is believed to occur by inhalation and primarily targets the lung with frequent dissemination to the central nervous system as well as a variety of other organs [12, 13].

*C. gattii* was first isolated from a leukemic patient in 1970 and described as a variant of *C. neoformans* [14]. *C. gattii* is closely related to *C. neoformans*, although its distribution is not global. *C. gattii* is typically restricted to tropical and subtropical geographical regions such as Australia, Brazil, and southern California [15, 16]. In the laboratory, these two cryptococcal species can be distinguished on the basis of their capsular serotype: *C. gattii* belongs to serotypes B and C, while *C. neoformans* belongs to serotypes A and D [17]. Like *C. neoformans*, *C. gattii* typically causes pneumonia and meningitis [15]. However, *C. gattii* appears to have a greater propensity to infect immune competent humans [18, 19].

For reasons that are not yet fully understood, *C. gattii* has acquired the ability to colonize new biogeoclimatic regions and is responsible for a recent outbreak of infection among humans and animals in the temperate climate of Vancouver Island, British Columbia (BC), Canada [20]. Between 1999 and 2006, 171 human cases of *C. gattii* infection were identified, including 8 fatalities [21]. Between 2002 and 2005, the incidence of *C. gattii* infection on Vancouver Island peaked at 36 cases/million people/year, a number that was significantly higher than the 0.94 cases/million people/year observed in endemic regions of Australia [22, 23]. The most common clinical manifestation of *C. gattii* infection on Vancouver Island was pneumonia [21]. Although the majority of human infections have been found on the east coast of Vancouver Island [23], clinical cases have also been reported on the BC mainland, Alberta, and the states of Oregon and Washington [22, 24, 25]. Given the ongoing spread of *C. gattii* infection on the Pacific coast of North America, it will be important for clinicians and laboratory scientists to remain vigilant for diseases that may be caused by this fungal pathogen.

## 3. Taxonomy

*Cryptococcus* is a largely polyphyletic genus that consists of at least 37 different species and belongs to the kingdom *Fungi*, phylum *Basidiomycota*, class *Tremellomycetes*, and order *Tremellales* [26–28]. The subclassification of this genus has been the subject of much debate and modification, particularly in response to the development of newer molecular typing methods [26, 27, 29]. The *C. neoformans* species complex was first classified according to structural variations of the extracellular polysaccharide capsule that are distinguished by agglutination assays with antigen-specific antibodies [30, 31]. Using this approach, *C. neoformans* was classified into four serotypes, A through D, in the 1950s and 1960s [30, 32]. The hybrid serotype AD is often considered to be a fifth serotype of the *C. neoformans* species complex, and rare hybrid serotypes between *C. neoformans* and *C. gattii* such as BD and AB have also been observed [33–37].

*C. gattii* was initially classified as a variety of *C. neoformans*, bearing the name *C. neoformans* var. *gattii* or var. *bacillispora* [15, 38]. Subsequently, *C. gattii* and *C. neoformans* were shown to have substantial differences in their biochemistry, ecology, epidemiology, and clinical manifestations (see review [15]) as a result of the divergence of serotypes A and D from serotypes B and C that occurred approximately 37 million years ago [15, 39]. Following a series of revisions to the classification of the *C. neoformans* species complex [11, 14, 17, 38, 40, 41], cryptococci are now divided into two major species, *C. neoformans* (serotypes A, D, and AD) and *C. gattii* (serotypes B and C). The sexual state of *C. gattii* is known as *Filobasidiella bacillispora* [38, 41].

The most recent classification of cryptococci was established by genetic typing using PCR fingerprinting, random amplification of polymorphic DNA (RAPD), amplified fragment length polymorphism (AFLP) analysis and multilocus sequence typing (MLST) [17, 23, 35, 37, 42–45]. Based on genetic diversity, cryptococci were divided into eight molecular types associated with distinct AFLP profiles. *C. neoformans* may be classified into VNI/VNII genotypes corresponding to AFLP1/1A/1B (serotype A), VNIII/AFLP3 (serotype AD), and VNIV/AFLP2 (serotype D), while *C. gattii* may be distinguished as VGI/AFLP4 (serotype B), VGII/AFLP6 (serotype B), VGIII/AFLP5 (serotype B or C), and VGIV/AFLP7 (serotype B or C). Interspecific hybrid serotypes BD and AB correspond to AFLP8 and AFLP9, respectively [35, 37]. The VNI and VGI genotypes are the most prevalent isolates of each species. The Vancouver *C. gattii* isolates have a VGII genotype that is further subtyped into VGIIa (major) and VGIIb (minor) [23]. A recent study employing MLST of one hundred and seventeen isolates confirmed previous observations that four monophyletic lineages exist within *C. gattii*. Based on these findings, it was suggested that these lineages should be considered as separate taxa, similar to the two monophyletic lineages within *C. neoformans* that correspond to varieties *grubii* and *neoformans* [45]. Another MLST study has indicated that although the Vancouver Island *C. gattii* strains have colonized a novel environment, they are not phylogenetically unique [46].

## 4. Ecology and Epidemiology of *C. gattii*

*C. gattii* is endemic in tropical and subtropical regions such as Australia where it is most commonly associated with eucalyptus trees, particularly *Eucalyptus camaldulensis* and *Eucalyptus tereticornis* [15, 47–49]. The fungus has also been found to grow on other tree species such as almond (*Prunus dulcis),* golden shower *(Cassia fistula),* and Douglas fir (*Pseudotsuga menziesii)* in Colombia, Brazil, and Vancouver Island, respectively [17, 47]. *C. gattii* has also been isolated from insect frass in Australia and a wasp nest in Uruguay [50, 51]. Other species in the Filobasidiella lineage, such as *Cryptococcus amylolentus*, *Tsuchiyaea wingfieldii*, and *Bullera dendrophila*, have also been isolated from insect frass [52–54]. In this context, the recent discovery of *C. gattii* in the Pacific Northwest is intriguing, since this temperate climate is characterized by mild and wet winters and warm and dry summers. On Vancouver Island, *C. gattii* has mainly been found on trees in the coastal Douglas fir (CDF) biogeoclimatic zone such as fir, cedar, and maple [23, 47]. The fungus has also been isolated from the air, freshwater, seawater, and upper layer of the soil of the BC mainland, the Gulf Islands, and Washington state [22, 23, 47].

Ecological and geographical differences exist among the different molecular types of *C. gattii*. A survey conducted in 16 countries revealed that the molecular types most commonly found in the clinic and environment were VGI and VGII [55]. Genotype VGIII has been found in the United States, Mexico, South America, Europe, India, Australia, and New Zealand, while VGIV has been isolated in Mexico, Colombia, Europe, South Africa, and India [44, 55–58]. A recent epidemiological survey across Europe showed that *C. gattii* clinical infection is rare in this continent, represented by only six of 535 serotyped *Cryptococcus* isolates [59]. In this study, the genotypes of the six European *C. gattii* isolates identified were not determined. In Australia, the *C. gattii* VGI genotype is the most common clinical or environmental isolate, while the VGII genotype is an infrequent cause of human or animal infection [15, 50]. A notable exception to this overall pattern is the Northern Territory of Australia that does not contain eucalyptus trees yet has the highest incidence of clinical *C. gattii* infection. Interestingly, most human cases in this region are due to VGII, suggesting that *C. gattii* has colonized a new environmental niche [60, 61]. This possibility is also supported by the fact that eucalyptus trees in the rest of Australia have only yielded the VGI genotype [62].

While the majority of *C. gattii* isolates in Australia's Northern Territory and on Vancouver Island are of the VGII genotype, a notable difference between the two regions is the remarkably limited diversity of the latter. Specifically, ninety five percent of the environmental and clinical samples on Vancouver Island are of the VGII genotype, and within this group 90% are of the major genotype VGIIa [47]. MLST studies have shown that the Vancouver Island VGIIa genotype is identical to two known *C. gattii* strains: the 1975 Seattle human isolate NIH 444 (also known as CBS 6956 and ATCC 32609) and the 1992 San Francisco environmental isolate CBS 7750 isolated from *E. camaldulensis* [63]. These studies have also demonstrated that the VGIIa isolates from Vancouver Island, the BC mainland, the Gulf Islands and northern Washington State are genetically identical [22]. These findings indicate that the major genotype VGIIa has existed in the Pacific Northwest for more than 30 years and it is hypothesized that VGIIa originated from sexual mating between VGIIb and another unknown parental strain [63]. Notably, VGI and VGII clinical isolates from Oregon differed from the Vancouver Island VGI and VGII samples at one or more of the loci that were analyzed [22]. The reason for this is not clear but may be explained by divergent evolution from a common genotype or the presence of distinct genetic isolates residing in the Pacific Northwest. The VGIIb minor genotype on Vancouver Island is identical to the unusually fertile Australian VGIIb clinical isolate NT-13, suggesting that the Vancouver Island isolate originated in Australia [63]. The VGI genotype is rarely found on Vancouver Island and it remains unclear whether it has actually colonized the environment, and the molecular types VGIII and VGIV have not yet been reported [23, 46]. Recent studies on *C. gattii* clinical and environmental isolates from South America have shown that VGII predominates over the other molecular types in both Colombia and Brazil as observed on Vancouver Island [47, 64, 65]. Meyer et al. have hypothesized that the Vancouver Island outbreak isolates originated in South America based on the finding of *α* mating-type cells within the Brazilian *C. gattii* VGII population and the fact that all Vancouver Island *C. gattii* isolates found to date have also been of the *α* mating type [63, 65, 66]. In addition, the VGII genotype has been present in Brazil longer than it has been on Vancouver Island [65]. In contrast, *C. gattii* VGII isolates (serotype B) in Colombia are mainly of the opposite a mating type, although large numbers are present in regions that have a temperate climate that is similar to Vancouver Island [64].

## 5. Virulence Factors

*C. gattii* and *C. neoformans* share many attributes that increase their ability to invade and survive in a host organism [92]. The main virulence factors identified in *C. gattii* to date include an outer polysaccharide capsule, melanin, mannitol, extracellular proteinase, products of the laccase pathway, superoxide dismutase, phospholipases, urease, and the STE12*α* transcription factor (a homologue of *Saccharomyces cerevisiae* STE12) that is present only in the *α* mating type (Table 1) [93]. Other properties of *C. gattii* contribute to its infectivity such as its ability to grow at physiological temperature [15], its tolerance of low pH and elevated salt levels [47], and its ability to switch capsular phenotype [91].

A carbohydrate-rich outer capsule that is composed primarily of glucuronoxylomannan (GXM) with smaller proportions of galactoxylomannan (GalXM) and mannoproteins is the major virulence factor for both *C. gattii* and *C. neoformans* [94]. The capsule may change in composition and size through a process called phenotypic switching (described further in the Notable Attributes section) and induces suppression of the host immune response by various mechanisms including the downregulation of cytokine and chemokine expression in dendritic cells (Table 1) [91, 95]. Kinetic studies have shown that complement component C3 binds less efficiently to *C. gattii* compared to *C. neoformans*, suggesting that *C. gattii* enhances virulence by preferential evasion of immune recognition [96]. Species-specific variation in the expression of other virulence factors may also play a role in their pattern of infectivity and organ dissemination. For instance, it was observed that extracellular proteinase production is lower in certain strains of *C. gattii* compared to a number of *C. neoformans* isolates, suggesting that *C. gattii* may less efficiently degrade proteins involved in tissue integrity and host immunity such as collagen, fibrin, complement, and immunoglobulin [79–82]. This finding may explain why *C. gattii* lesions are often more circumscribed compared to *C. neoformans*, a characteristic that could also reduce local and systemic dissemination [82, 97]. Similarly, in vitro experiments with a variety of *C. neoformans* and *C. gattii* isolates from South America have shown that *C. neoformans* has increased urease production relative to *C. gattii* [98]. Intratracheal administration of the highly virulent *C. neoformans* H99 (serotype A) to mice suggests that urease may aid in the dissemination of the fungus from the lung to the central nervous system, although the exact mechanism is unknown [88]. It is also interesting to note that virulence factors are involved not only in pathogenesis but also in commensalism. For instance, *C. gattii* was observed to share an endophytic relationship with decaying wood of both eucalypt and noneucalypt trees, where the laccase enzyme system appears to play a role in digestion of lignin [15, 50].

Given that *C. neoformans* and *C. gattii* appear to share many of the same virulence factors, it is intriguing that *C. gattii* most commonly infects immune competent individuals while *C. neoformans* primarily infects the immune compromised host. One explanation for this observation is that immune compromised individuals simply have more environmental exposure to *C. neoformans* compared to *C. gattii* [5]. Furthermore, the contribution of host genetic background to resistance against cryptococcal infection is even less well understood. Interestingly, certain groups of individuals such as the Australian aboriginal population may be predisposed to *C. gattii* disease [62]. Modern molecular dissection of a microorganism's virulence factors may allow researchers to better understand the genetic mechanisms underlying virulence. A recent example of this approach used systematic targeted gene deletion in *C. neoformans* with comprehensive profiling of individual mutants in an animal model [99]. A similar large-scale strategy may be required to clearly delineate the pathogenesis of *C. gattii* and may in turn stimulate the development of novel therapeutic strategies.

## 6. Notable Attributes of *C. gattii*

Phenotypic switching and same-sex mating are two interesting attributes that contribute to the virulence of *C. gattii*. The term phenotypic switching refers to an adaptive mechanism characterized by structural modifications of the extracellular capsule and cell wall [91]. This phenomenon occurs infrequently in vitro or during chronic infection and a switch to a more mucoid form is associated with greater virulence in *C. neoformans* [91, 100]. Reversible phenotypic switching between a smooth and mucoid variant of *C. gattii* strain NP1 has also been identified [100]. Shortly after infection, the mucoid form of NP1 is most commonly observed; however, a subsequent switch to the smooth form characterized by reduced capsular polysaccharide allows for easier penetration of the blood-brain barrier and dissemination to the brain [91]. Evaluation of survival and fungal burden in BALB/c mice following intravenous or intratracheal infection with either *C. gattii* NP1 phenotypic variant demonstrated increased virulence of the mucoid form. In this study, both mucoid and smooth variants were found in the lung homogenates while only the smooth variant was found in the brain homogenates. In terms of the immune response, infection with the smooth variant elicited a greater inflammatory response as characterized by lymphocyte and monocyte infiltration and gave rise to smaller cryptococcomas than the mucoid variant [91].

In contrast to most Australian *C. gattii* isolates, it was demonstrated that all Vancouver Island isolates belong to the *α* mating-type and are unusually fertile [63, 66]. The mechanism by which cryptococcal cells of an identical mating type replicate is not known. However, it has been suggested that *C. gattii* undergoes same-sex mating, a process that has been studied in *C. neoformans* [63]. Upon nutrient limitation, *α* haploid cells can undergo sexual recombination via fruiting, a process by which haploid cells fuse, chromosomal reassortment and recombination occur, followed by meiosis and sporulation [66, 101]. Same-sex mating between two *α* cells (rather than an *α* and an a cell) may confer a survival advantage and could explain why all Vancouver isolates are of the *α* mating type [101]. Both traditional and same-sex mating in *C. neoformans* have been observed in the laboratory but not in nature [63, 66, 101, 102].

## 7. Animal Models of *C. gattii* Infection

A limited number of animal model studies of *C. gattii* infection have been reported in literature. One group has investigated the survival of A/J inbred mice following intranasal infection with different Vancouver Island *C. gattii* molecular types. In this report, the major genotype VGIIa appeared most virulent (20% survival at 15 days post-infection), the minor genotype VGIIb was avirulent (100% survival at 55 days post-infection), and the VGI genotype was similar in virulence to VGIIa [63]. These findings are consistent with recent clinical experience that has shown VGIIa to be the most common isolate from patients [21, 22, 24, 103]. 

Further insights into disease pathogenesis have been derived through animal studies using *C. gattii* isolates that were not obtained from Vancouver Island. One study compared mouse and human pulmonary inflammatory responses to intratracheal infection with 10 different Australian isolates of *C. gattii* [104]. In BALB/c mice, 6 isolates did not elicit any inflammatory response, 3 provoked a minimal response, and 1 resulted in a strong host inflammatory response that took several weeks to develop. The peak inflammatory response was observed 5 weeks after infection and was characterized by foamy macrophages, lymphocytes, poorly defined granulomas containing some giant cells as well as *C. gattii* yeast, and destruction of lung tissue. In humans, variable pulmonary pathology has been detected including poorly formed granulomas containing intracellular *C. gattii*, lymphocytic interstitial pneumonitis, tissue necrosis, and fibrosis. In both species the local lymphocyte pool consisted largely of T-cells with a 2:1 ratio of CD4 to CD8 cells in humans [104].

A second study examined the pathology seen in BALB/c mice following systemic infection with the clinical isolate *C. gattii* 9714 (CBS 6996, serotype B, VGIII/AFLP5) under different experimental conditions [105, 106]. It was observed that both immune competent and hydrocortisone-treated mice developed *C. gattii* disease, contrasting with the apparent predilection of immune competent humans to this infection. At high doses of *C. gattii*, immune suppressed mice were more likely to develop severe disease compared to immune competent mice. SCID mice that lack T- and B-lymphocytes were more susceptible to infection relative to wild-type BALB/c, suggesting a protective role for these cell types [105]. Comparative studies showed that *C. neoformans* 9759 (a serotype A clinical isolate) has increased virulence compared to *C. gattii* 9714 after systemic infection of BALB/c mice, regardless of the immune state of the animal. Specifically, *C. neoformans* 9759 infected the brain and lungs of BALB/c mice, while *C. gattii* 9714 affected the lungs and skin. Ulcerative lesions on the tail due to intravenous *C. gattii* infection were more common in immune competent mice, while rectal prolapse was more common in immune suppressed mice. This study also demonstrated that infection by either *C. neoformans* 9759 or *C. gattii* 9714 may cause gastrointestinal pathology, although its incidence was rare [105].

A third study examined the pathogenesis of five *Cryptococcus* isolates administered to BALB/c mice via the intraperitoneal route [107]. The isolates included *C. gattii* GR52 and GR56 (both serotype B), from immune competent goats that died of pneumonia in Spain; *C. gattii* B4506 (serotype B), an Australian environmental isolate known to be highly pathogenic in mice; *C. gattii* I-682 (serotype C), from a Colombian native almond tree; and *C. neoformans* GR297 (serotype D), from an AIDS patient in Spain with meningitis. Two of five mice infected with *C. neoformans* GR297 developed liver and peritoneal abscesses and one mouse died after four weeks. Fungal cultures of spleen, liver, kidney, testes, lung, and brain were done for each group and were positive in at least one organ for GR52, GR297, GR56, B4506, and I-682 in 80%, 77%, 70%, 70%, and 33% of the cases, respectively [107]. Based on the frequency of cryptococcal growth in the organ cultures, all fungal isolates with the exception of the serotype C sample I-682 displayed similar virulence in BALB/c mice. Among the organs tested, the spleen was most often positive for *Cryptococcus* (91%), followed by the liver (75%), kidney (75%), testes (71%), lung (62%), and brain (29%). *C. gattii* GR52, the cause of a pneumonia outbreak in goats, was also isolated from the lungs of all BALB/c mice studied, suggesting that it has a particular tropism for lung tissue. Pathologic findings associated with infection of the brain included areas of spongiosis in the white matter with an abundance of encapsulated yeasts in the basal ganglia while infected lungs showed encapsulated yeasts within alveoli, peribronchial vessels, and interalveolar spaces. Interestingly, inflammation was not observed in either organ among infected mice [107].

These studies show that the route of experimental cryptococcal infection is a major determinant of the site of disease. In general, intravenous infection directly induces systemic disease, whereas inhalation of the fungus via the airway mimics the natural route of infection and leads to primary disease in the lung [108]. For example, in the second study both the cutaneous and gastrointestinal pathologies may have been a direct result of *C. gattii* administration through the tail vein, and significant involvement of the visceral organs and peritoneum in the third animal study may have resulted from intraperitoneal inoculation of the fungus. Nonetheless, the tissue tropism observed in humans is similar to that seen in these mouse models [13, 15, 21]. In addition to the route of infection, these studies also confirm that other variables such as the *C. gattii* isolate, infectious dose, and immune status of the host also play a role in the virulence of the fungus and the severity of infection [15].

## 8. Veterinary Cases

*C. gattii* has the potential to infect a wide range of animals throughout the world, including wild, farm, domestic, and aquatic animals, along with a variety of birds. This broad host range was observed during the recent outbreak in British Columbia [23, 109, 110]. In order to study the epidemiology of veterinary cases, diagnostic methods to test for infection in animals generally consist of serum and/or tissue sampling and nasal swabbing [28, 109, 111–113]. A recent study examined the characteristics of *C. gattii* infection in domestic animals in southwestern BC, involving 78 feline and 51 canine cases [28]. It was observed that cats were 4.4 times more likely than dogs to be positive for *C. gattii* infection, and that all cases in dogs and 50% of cases in cats were due to *C. gattii* serotype B [111]. In cats, the median age at diagnosis was 7.3 years, whereas in dogs it was 2.3 years [28]. The tissue tropism of *C. gattii* disease differs slightly in cats and dogs. Among canines, the affected organs included the respiratory system (52%), central nervous system (42%), and the skin or gastrointestinal tract (6%), while in felines 56% were respiratory, 26% involved the central nervous system, and 19% were dermal with no cases of gastrointestinal involvement [28].

Another study was carried out on Vancouver Island to determine whether wild animals were infected with *C. gattii*. Of the 91 animals that underwent swabbing of the nasal cavity, two eastern grey squirrels were found to be positive for *C. gattii*. This number is similar to the incidence of infection in asymptomatic companion animals; specifically 1.1% of dogs, 4.3% of cats and 1.5% of horses were positive for *C. gattii* [109]. Subclinical *C. gattii* infection in dogs and cats does not necessarily progress to clinical disease. In some cases it may be cleared while in other cases it may persist or develop into more severe disease [115]. It has also been suggested that infected wild animals may be an important vector for the spread of *C. gattii* from Vancouver Island to geographic regions that do not harbor the organism and that veterinary cases may also be a reliable sentinel of human disease [109]. The latter assertion is based on the observation that *C. gattii* infection was identified in animals prior to the human outbreak that began on Vancouver Island in 1999 and that nearly twice as many animals appeared to be infected with *C. gattii* compared to humans between 1999 and 2003 [116]. These observations also highlight a potential role for animals in transmission of infection to humans. While zoonotic transmission of *C. gattii* has not been reported to date, transmission of *C. neoformans* from pet birds to humans has been documented, and possible human-to-human transmission has also been described [117–119]. In addition to the veterinary cases in the Pacific Northwest, outbreaks of *C. gattii* involving goats in Spain [120] and psittacine birds in Brazil [112] have also been observed.

## 9. Human *C. gattii* Infection

*C. gattii* may cause mild to severe clinical disease in the apparently healthy as well as immune compromised host. Like other cryptococcal species, *C. gattii* enters the human host through the inhalation of airborne propagules and targets the lung as a primary site of infection [22, 108]. In some cases dissemination via the bloodstream may occur, most commonly to the central nervous system (CNS) with occasional spread to other organs such as skin, eye, and prostate [13, 15, 82]. The predominant manifestations of *C. gattii* infection involve the lungs in the tropical climate of Australia (66% pulmonary) as well as the temperate climate of Vancouver Island and its surroundings (75% pulmonary) [21, 60].

A study performed by the British Columbia (BC) Cryptococcal Working Group at the University of British Columbia reviewed the clinical aspects of 171 individual human cases of *C. gattii* infection on Vancouver Island from 1999 to 2006 [21]. It was determined that the mean age at diagnosis was 59 years, with a range of 2 to 92 years. Males (56%) appeared to be slightly more susceptible to infection than females (44%) [21]. The clinical patterns of *C. gattii* infection were pulmonary (75%), neurological (8%), combined (9%), and unknown (8%). Of those patients with combined disease, the main sites involved the lung and CNS, and less commonly the skin and lung, or skin and CNS. Death was a rare outcome of *C. gattii* infection on Vancouver Island with 8 fatalities from 1999 to 2006, or approximately one death per year. Of the deceased patients, five presented with both pulmonary and CNS pathology, three had other underlying comorbidities, and one had an adverse reaction to therapy. The mean age of the deceased patients was 61 years (range from 26 to 87) [21].

A summary of the most recent case reports associated with the Pacific west coast is presented in Table 2. Among the first 8 patients, three patients resided in British Columbia, two patients resided in Oregon State, one resided in Washington state, one resided in Alberta, and one patient was a tourist from Denmark visiting Vancouver Island [22, 24, 25, 103]. The place of residence of patients 9 to 12 was not specified, although they represented patients discharged from Vancouver Island hospitals [114]. Six of the cases were either not directly exposed to *C. gattii* on Vancouver Island or were exposed many years earlier. This suggests that another source of *C. gattii* may exist outside of Vancouver Island, or that the fungus may have been transiently present in other geographic regions due to dispersal mechanisms [22, 121]. Also, the fact that in two cases infected individuals had traveled to Vancouver Island 4 and 14 years before developing symptoms raises the possibility that *C. gattii* may remain latent in the body and reactivate at a later time [5].

In the BC Cryptococcal Working Group study, the pulmonary symptoms most commonly observed in the 171 cases were cough and dyspnea, while the main symptom involving the CNS was headache [21]. Similarly, the usual symptoms in the Pacific west coast case reports were cough and shortness of breath, although some patients developed nausea, fever, headache, muscle pain, and loss of appetite. It is important to recognize that the clinical presentation of *C. gattii* infection may be quite subtle. For example, patients 3 and 9 were asymptomatic while patient 6 experienced only a nonspecific cough [22, 24, 114]. The most common pathology observed in these patients was nodules of the lung in the form of cryptococcomas. Interestingly, patient 7 presented with pulmonary ground-glass opacities, a pattern that is quite distinct from circumscribed nodules and rarely observed in the clinic [25]. The specific pulmonary pathology for patient 4 was not reported [22]. Meanwhile, CNS pathology was documented in four patients: patient 1 had cerebral cryptococcomas and patients 5, 7, and 12 presented with meningitis [22, 25, 114].

All 171 *C. gattii* cases from the BC Cryptococcal Working Group study were caused by *C. gattii* serotype B [21]. This finding is consistent with the fact that serotype B exists at a much higher frequency in the environment compared to serotype C. In fact, the identification of *C. gattii* serotype C in the environment had not been described prior to 1998 [50, 122]. It also appears that the *C. gattii* major genotype VGIIa is highly associated with human disease: 82% of the cases were VGIIa, while the others were VGIIb (9%) and VGI (6%) [21]. Similarly, 5 of the 12 case reports from the Pacific west coast were VGIIa, one was VGIIb, and another was VGI, while the rest were unspecified. Apart from place of residence or travel, the main risk factors associated with the development of *C. gattii* disease were smoking (50%), oral steroid use (30%), invasive cancer (24%), and chronic lung disease (13%). HIV infection (4%) and receipt of an organ transplant (3%) were considered less common risk factors for the development of *C. gattii* disease in this study population [21]. Examination of the case reports shows a similar pattern; 4 of the 12 cases had a current or past history of smoking, and 7 of the 12 patients suffered from chronic disease and/or cancer, had undergone recent surgery, or had been exposed to corticosteroids.

Thus far, the median incubation time of *C. gattii* on Vancouver Island has been estimated to be 6-7 months with a range from 2 to 11 months [123]. One exception is the relatively short incubation period of six weeks that was observed in the Danish tourist visiting Vancouver Island [103]. The potentially long time frame from exposure to symptoms may impede the diagnosis of *C. gattii* infection. It is likely that the clinical incubation period depends on several variables such as differences in host immunity and the intensity of *C. gattii* exposure.

## 10. Microbiology

Laboratory testing is essential to diagnose *C. gattii* infection and generally requires analysis of tissue or fluids from infected sites such as cerebrospinal fluid, bronchial washings, blood, and urine [12]. Light microscopy is an efficient method for the rapid diagnosis of cryptococcosis. For this technique, fluid samples are usually stained with India ink while tissue samples may be stained with hematoxylin and eosin (H&E), mucicarmine, or other stains [12, 20, 91]. In both cases, cryptococcal cells appear round to oval in shape, surrounded by a wide capsule. Infrequently, one may visualize nonencapsulated cryptococci under the microscope [12]. In the laboratory, both *C. neoformans* and *C. gattii* readily grow as round cream-colored mucoid colonies on Sabouraud dextrose agar, a selective medium that is widely used for the isolation of yeast [12]. Alternatively, malt extract agar may be used to selectively isolate fungi [12, 47]. Differential microbial media such as Staib agar or birdseed agar may be used to distinguish dark brown colonies of *C. neoformans* or *C. gattii* from other fungi [47, 124]. Further differentiation of cryptococcal species can be accomplished on L-canavanine-glycine-bromthymol blue (CGB) agar; *C. gattii* colours the medium blue while *C. neoformans* does not (medium remains yellow) [125]. Though not required for clinical management, the fungus may be viewed in its sexual state by growing the cells on V8 medium in the dark followed by fixation and staining to visualize the hyphae, nuclei, and septa [101]. Finally, it is possible to determine the exact serotype of a cryptococcal isolate using a slide agglutination assay although commercial kits for this purpose are not currently available [22, 25].

 In vitro activity of antifungal agents may be helpful in guiding the clinical management of severe cryptococcal infection including meningoencephalitis [126]. A slower clinical response of *C. gattii* to antifungal therapy has been attributed to serotype-specific differences in antifungal activity though there are no consistent or predictable data to support this hypothesis [127, 128]. To evaluate this possibility further, a recently published study compared the susceptibility of 86 *C. neoformans* and 42 *C. gattii* isolates to various antifungal drugs including amphotericin B, flucytosine, fluconazole, posaconazole, voriconazole, and isavuconazole [129]. The major finding was that all antifungal agents tested retained activity against all cryptococcal isolates with the newer azoles exhibiting greater potency compared to fluconazole and flucytosine. In this report, no significant differences in drug potency were observed for *C. gattii* serotypes B or C compared to other serotypes.

## 11. Spread of the Vancouver Island *C. gattii* Outbreak

The colonization of *C. gattii* on Vancouver Island, and possibly other adjacent regions, indicates that this fungus has the ability to adapt to new environmental conditions. It has been suggested by Kidd et al. that the temperate climate of Vancouver Island may provide a favorable niche for the survival and dispersal of *C. gattii* [23]. Global warming has also been proposed to favor *C. gattii* colonization of new geographic regions [23]. Studies in Colombia have also suggested a correlation between climate and distribution of cryptococcal serotypes A to C. Specifically, differential tolerance of climatic and possibly other environmental conditions by individual serotypes may affect their geographic and ecological distribution [37, 130]. The modes of dispersal of *C. gattii* have been studied on Vancouver Island. Some trees in the region show intermittent positivity, suggesting the movement of fungal spores [121]. High *C. gattii* levels exist in public areas such as park entrances, parking lots, and beaches despite the fact that these areas are characterized by low tree density [47, 121]. A significant amount of *C. gattii* dispersal appears to be mediated by human activity, including contact with footwear and car wheel wells, deforestation, and gardening [121]. Avian and perhaps other animal migrations, as well as insect vectors such as beetles and caterpillars [15], may also contribute to dispersal [121].

Has *C. gattii* colonized other regions on the Pacific west coast? It is known that *C. gattii* (serotype C) was isolated from a eucalyptus tree in San Francisco in 1992, but since then *C. gattii* has not been found in that environment. In order to determine whether *C. gattii* has spread to the United States, Fraser et al. investigated possible colonization on the San Juan Islands, the closest U.S. region to Vancouver Island [131]. The San Juan Islands also exhibit a temperate climate zone that is similar, but not identical, to the CDF biogeoclimatic zone of Vancouver Island. Although other fungi of the phylum *Basidiomycota*, namely *Cryptococcus* *laurentii* and *Cryptococcus cellulolyticus*, were isolated, *C. gattii* was not detected. This suggests that *C. gattii* may not have yet colonized the San Juan Islands, or dispersal of the fungus is impeded by a geographical barrier that is the Juan de Fuca strait [131]. In a recent study by MacDougall et al., *C. gattii* was isolated from environments outside of Vancouver Island including the British Columbia mainland, the Gulf islands, and Washington state, but no positive environmental samples were found in Oregon [22]. In the clinic, *C. gattii* has been isolated in Seattle (1975), Vancouver Island, and the British Columbia mainland, and most recently in Oregon, Washington, and Alberta [22–25, 63].

## 12. Conclusion

The recent outbreak of *C. gattii* infection in the Pacific Northwest highlights the fact that this fungus is an important emerging pathogen that can adapt to new potential environmental niches. *C. gattii* isolates found on Vancouver Island appear to be hypervirulent, as the number of infected individuals per million people per year between 2002 and 2005 was more than 36 times higher on Vancouver Island compared to endemic regions such as Australia [22, 23]. It is in the interest of scientists and clinicians alike to better understand the pathogenesis of *C. gattii* disease in order to discover effective prevention and treatment strategies, including measures to limit human exposure to this pathogenic fungus. Employing *C. neoformans* as a model organism to understand the disease-causing potential of *C. gattii* is no longer sufficient since fundamental disparities such as differences in host tropism and the expression of certain virulence factors exist between the two species.

## Figures and Tables

**Table 1:** *C. gattii* virulence factors and their functions.

Factor	Function
Capsule and its associated polysaccharides	Evasion of phagocytosis [67]
	Reduction of antigen presentation [68]
	Reduction of cytokine production [69]
	Induction of suppressor T-cells which inhibit cell-mediated immunity [70]
	Inhibition of T-cell responses by GXM [31]
	Inhibition of leukocyte migration into inflammatory sites by GXM [31, 71]

Melanin	Protection against UV radiation [72]
	Protection against oxygen and nitrogen free radicals [73, 74]
	May contribute to central nervous system tropism [6]
	Contributes to negative cellular charge [75]

Mannitol	Suggested increase in intracranial pressure [76]
	Protection against stress [77]
	Protection against oxygen free radicals [78]

Extracellular protease	Proteolytic activity [79]
	May contribute to degradation of proteins involved in tissue integrity and host immunity [79–82]

Products of laccase pathway	Diphenol oxidation [83]
	Synthesis of melanin [84]
	Degradation of wood lignin [15]

Superoxide dismutase	Protection against oxidative stress [85]
	Protection against oxidative burst produced by immune effector cells [85]

Phospholipases	Tissue invasion via degradation of mammalian membrane lipids and lung surfactant [15, 86]

Urease	Exact function is unknown [87]
	May aid in transfer of *Cryptococcus* to central nervous system [88]

STE1*α* transcription factor (in cells of *α* mating type)	Upregulation leads to synthesis of diphenol oxidase (which is a laccase) [89, 90]

Growth at physiological temperature (37°C)	Survival and persistence in the host [15]

Tolerance of low pH	Survival and persistence in the environment [47]

Tolerance of elevated salt	Survival and persistence in the environment [47]

Phenotypic switching	Change in capsule size—mucoid variant more virulent, smooth variant suggested to be able to cross blood-brain barrier [91]

**Table 2:** Summary of case reports of *C. gattii* infection.

	Age (sex)	Residence	Exposure	Health conditions	Symptoms	Pathology (molecular type)
**1** [22]	47 (M)	BC mainland (on coast north of VI)	Yard/landscaping work at residence	Chronic hepatitis C, drug addiction	Cough, chills, night sweats, nausea, muscular pain, headache, appetite loss, stiff neck	Lung/brain nodules (VGI)

**2** [22]	48 (F)	BC (lower mainland)	Deforestation near residence, yard work, last visited VI 4 years ago	None	Dyspnea, fever, chills, headache, night sweats, appetite loss, nausea, muscular pain	Lung mass (VGIIa)

**3** [22]	73 (F)	BC	Little outdoor exposure, last visited VI 14 years ago	Chronic renal failure, lung disease, breast cancer, hip surgery	None	Lung nodule (VGIIa)

**4** [22]	59 (M)	OR	No travel out of OR in last year	Scarred lung tissue (due to occupation)	Cough, dyspnea, fever, chills, nausea, weight loss, muscular pain	Not specified (VGIIa)

**5** [22]	87 (M)	OR	Travel to parts of OR, WA & CO	Chronic lymphocytic leukemia (oral steroid)	Fever, weight loss, appetite loss	Meningitis (VGIIb)

**6** [24]	74 (M)	WA (Puget Sound)	Travel to south CA and HI in last year	Large granular lymphocytic leukemia (steroid and hormone)	Cough	Lung nodule (VGIIa)

**7** [25]	45 (F)	Alberta	Holidays spent on southeast shore of VI, park visits	None	Headache, blurred vision, photosensitivity, nausea, vomiting, cough	Lung opacities, airspace disease, meningitis (not specified)

**8** [103]	51 (M)	Denmark	Travel to Victoria and VI east coast for 7 days, garden visits	Psoriatic gout (nonsteroidal anti-inflammatory drug)	Chest pain, fever, cough, dyspnea	3 large nodular infiltrates (VGIIa)

**9** [114]	54 (M)	Not specified	Not specified	Bradycardia, acute myocardial infarction, smoker	None	Lung opacity (not specified)

**10** [114]	62 (M)	Not specified	Not specified	Previous smoker	Chest pain	Lung nodules (not specified)

**11** [114]	69 (F)	Not specified	Exposure to tuberculosis	Previous smoker, total colectomy due to ulcerative colitis	Cough, dyspnea	Cavitary lesion (not specified)

**12** [114]	65 (M)	Not specified	Occupational (painter)	Previous smoker, mild recurrent hemoptysis	Not specified	Lung opacity, meningitis (not specified)

*Legend*: BC: British Columbia, CA: California, CO: Colorado, HI: Hawaii, OR: Oregon, VI: Vancouver Island, WA: Washington.
